# Distinct Patterns of Colorectal Peritoneal Metastases at Initial and Repeat Cytoreductive Surgery

**DOI:** 10.1002/wjs.70435

**Published:** 2026-06-03

**Authors:** Mina Sarofim, David L. Morris

**Affiliations:** ^1^ Liver and Peritonectomy Unit St George Hospital Sydney Australia; ^2^ Innovation, Surgical Teaching and Research (iSTAR) Unit Liverpool Australia; ^3^ School of Medicine University of Sydney Sydney Australia

## Abstract

Colorectal cancer rates are continually increasing in Western countries exceeding over 3 million new cases annually. Hematogenous spread to the liver is the most common metastatic route, followed by transcoelomic dissemination to the peritoneum. Colorectal peritoneal metastases (CRPM) affect 5%–8% at the time of initial diagnosis and a further 10%–20% who develop recurrence. Cytoreductive surgery (CRS) is the gold‐standard treatment for selected patients to achieve locoregional control.

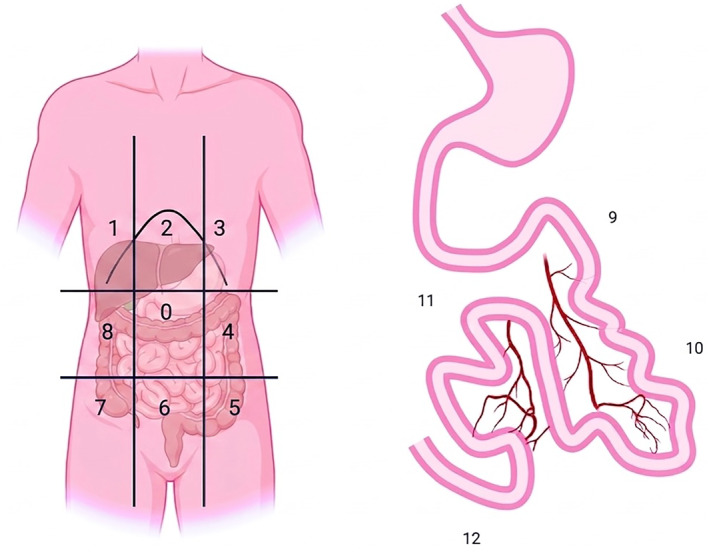

## 1 Introduction

Colorectal cancer rates are continually increasing in Western countries exceeding over 3 million new cases annually [1]. Hematogenous spread to the liver is the most common metastatic route, followed by transcoelomic dissemination to the peritoneum. Colorectal peritoneal metastases (CRPM) affect 5%–8% at the time of initial diagnosis and a further 10%–20% who develop recurrence. Cytoreductive surgery (CRS) is the gold‐standard treatment for selected patients to achieve locoregional control. An even more selective subset undergo repeat CRS (rCRS) for recurrent CRPM to prevent disease‐related morbidity, such as ascites, obstruction, or enteric fistulae, extending median survival by 20–62 months [2].

**FIGURE 1:**
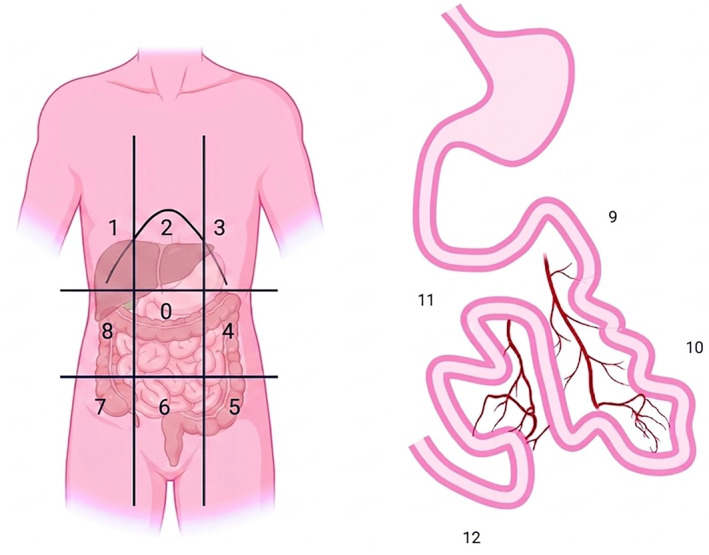
The 13 regions to determine peritoneal cancer index.

The peritoneal cancer index (PCI) developed by Sugarbaker (Figure 1) remains the most validated tool to quantify disease burden and guide patient selection for CRS [3]. However, no prior study has compared intraoperative PCI patterns region‐by‐region between initial CRS (iCRS) and rCRS. Determining whether recurrent CRPM exhibits a distinct more localized distribution could inform surgical planning, surveillance strategies, and potentially broaden selection criteria for rCRS. Therefore, this study aims to compare intraoperative PCI scores and regional involvement between iCRS and rCRS for CRPM, identifying specific patterns that may reflect altered tumor biology.

## 2 Methods

A prospective study was conducted at a tertiary CRS unit in Sydney Australia between January 2021 and December 2023. Eligible patients were adults (≥ 18 years) with metachronous CRPM undergoing either iCRS or rCRS after surgical oncology multidisciplinary review. All underwent hyperthermic intraperitoneal chemotherapy (HIPEC) with Mitomycin‐C for 90 min. Primary outcomes were total intraoperative PCI score and number of PCI regions involved recorded by the same lead surgeon. Statistical analyses were performed using SPSS v29 (IBM, USA); continuous variables were summarized as mean (± SD) or median (range) as appropriate. Comparisons between iCRS and rCRS were made using t‐tests, Mann–Whitney U tests, or χ^2^ tests; significance was defined as *p* < 0.05. The research protocol is in accordance with the Declaration of Helsinki, obtained ethics approval from the local health district and preregistered on Open Science Framework registry (https://osf.io/a35hk).

## 3 Results

A total 112 consecutive patients were included: mean age 56 years; 57% male; and 94 (84%) underwent iCRS and 18 (16%) rCRS (Table 1). Patients with rCRS were significantly younger and had lower median PCI scores compared with iCRS. Similar rates of complete cytoreduction (CC‐0) were achieved in both groups.

**TABLE 1:** Comparison of initial and repeat patients with CRS.

	Initial CRS	Repeat CRS	*p*‐value
Number	94	18	—
Age mean [SD]	56 [14]	53 [13]	< 0.001
Neoadjuvant chemotherapy *n* [%]	58 [62]	8 [44]	0.335
PCI median [R]	8 [3–39]	6 [2–12]	0.003
Regions without disease^a^ median [R]	8 [1–12]	11 [7–12]	0.039
CC‐0 *n* [%]	82 [87]	14 [78]	0.458
Length of stay mean days [SD]	22 [15]	13 [4]	0.002
Complication^b^ *n* [%]	32 [34]	4 [22]	0.487
Mortality *n*	0	0	—

Abbreviations: CC‐0, complete cytoreduction; CRS, cytoreductive surgery *n*, number; PCI, peritoneal cancer index; R, range; SD, standard deviation.

^a^ Out of total 13 PCI regions.

^b^ Clavien–Dindo Classification Grade III or IV.

**FIGURE 2:**
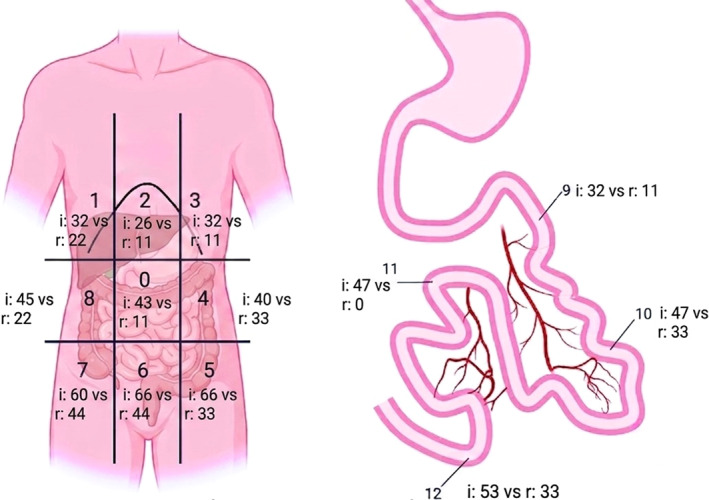
Percentage of patients in each group with disease in each of the 13 regions, i, initial CRS; r, repeat CRS.

As shown in Figure 2, the number of PCI regions involved was significantly fewer in rCRS (*p* = 0.039). Region‐specific analysis revealed 100% absence of proximal ileal disease (region 11) in rCRS compared with 24/94 (26%) in iCRS (*p* = 0.036). Length of stay was significantly shorter for rCRS, with comparable complication and mortality rates.

## 4 Discussion

This first prospective region‐by‐region PCI comparison demonstrates that rCRS for CRPM is characterized by a more localized disease burden, fewer peritoneal regions involved, and notably no proximal ileal involvement. It is also unknown whether such a distribution is purely preoperative selection bias (the key limitation) or a more complex interplay of systemic therapy, mechanical restriction of dissemination by initial HIPEC/postoperative adhesions, tumor genomics, immune evasion mechanisms, or metabolic biomarkers [4, 5].

Despite the modest sample size, rCRS was associated with shorter length of stay and similar complication rates which supports a more liberal approach to patient selection to offer repeat procedures to a wider cohort of appropriately selected patients. Future studies should integrate PCI mapping with molecular profiling and neoadjuvant chemotherapy [6] to better predict recurrence distribution and optimize both surgical and systemic treatment strategies. Multicenter collaborations to expand the sample size and the use of propensity score matching to reduce confounding factors may also be incorporated into future research comparing similar cohorts.

## Author Contributions

**Mina Sarofim:** conceptualization, writing – review and editing, investigation. **David L. Morris:** conceptualization, resources.

## Funding

The authors have nothing to report.

## Conflicts of Interest

The authors declare no conflicts of interest.

## Assistance

The authors have nothing to report.

## Registration

Registered on Open Science Framework registry (https://osf.io/a35hk).

## Data Availability

Dataset generated is available upon reasonable written request to the corresponding author.
